# Field dependent-independent cognitive style as a predictor of malevolent creativity: a multifaceted approach

**DOI:** 10.3389/fpsyg.2025.1502823

**Published:** 2025-07-02

**Authors:** Pierpaolo Zivi, Marco Giancola, Raffaella Nori, Laura Piccardi, Simonetta D'Amico, Massimiliano Palmiero

**Affiliations:** ^1^Department of Communication Sciences, University of Teramo, Teramo, Italy; ^2^Department of Biotechnological and Applied Clinical Sciences, University of L'Aquila, L'Aquila, Italy; ^3^Department of Psychology, University of Bologna, Bologna, Italy; ^4^Department of Psychology, “Sapienza” University of Rome, Rome, Italy; ^5^San Raffaele Cassino Hospital, Cassino, FR, Italy

**Keywords:** creative process, creative product, creative behavior, field dependent-independent cognitive styles, ethics, black humor, divergent thinking

## Abstract

**Introduction:**

Research interest in the intentionally harmful use of creativity, also known as malevolent creativity, is growing rapidly. However, the cognitive and individual underpinnings of malevolent creativity are still unclear. By employing a multifaceted approach, field dependent-independent cognitive style (FDI) was investigated as a potential individual component that may predict the likelihood of generating malevolent ideas (i.e., creative process), products (i.e., creative production), and engaging in original but malicious acts (i.e., creative behavior).

**Methods:**

Based on the literature associating FDI with general creativity, the hypothesis that field-independent individuals were more prone to the three facets of malevolent creativity was tested after controlling for demographic factors, social desirability, state mood, and ethical positions (idealism and relativism). Malevolent creativity was assessed through a divergent thinking task (process), a solicitation to produce black humor by a cartoon captions task (product), and a self-reported questionnaire concerning everyday creative acts (behavior).

**Results:**

The results showed that higher levels of field independence predicted malevolent creative process and product, whereas no differences emerged in creative behavior.

**Discussion:**

By partially replicating the evidence connecting FDI and creativity, the present study suggests that general and malevolent creativity share common grounds. Future studies are needed to overcome the current limitations in assessing malevolent creativity in everyday settings and to investigate further commonalities and differences between the two uses of creativity.

## 1 Introduction

Creativity is typically conceptualized as the ability to generate novel and appropriate outcomes (Runco, 2025). Despite this basic definition, creativity is commonly viewed from a positive standpoint, regardless of the social valence and morality of the products. In this regard, extensive research has drawn attention to the “benevolence bias” that invests the notion of creativity in both common sense and scientific literature (Cropley et al., 2008; Cropley and Cropley, 2019). Indeed, creativity can also lead to unfavorable outcomes and cause harm to others, either unintentionally (*negative* creativity) or deliberately (*malevolent* creativity). The interest in investigating the underpinnings of the harmful use of creativity was motivated not only by its involvement in terrorism and criminal acts but also in more common unethical situations, such as harassment and deception.

Theoretical models emphasized the multifaceted nature of creativity, distinguishing between creativity as a process and creativity as a product, among other dimensions (e.g., Rhodes, 1961). This distinction suggests that malevolent creativity can also be defined in various ways. The process dimension concerns the cognitive operations underlying creative thinking, while product refers to the tangible or intangible outcomes of such creative processes. The research explored creativity mainly through the lens of divergent thinking (DT), which defines the ability to generate many different unconventional ideas (Guilford, 1967; Giancola et al., 2023a). DT represents a long-standing and largely used esteem of an individual's creative potential (Runco and Acar, 2012), which can be appreciated in terms of fluency (number of appropriate ideas), flexibility (categorical shifts or number of categories encompassing the relevant ideas), persistence (number of ideas provided within a single category, that is ideational search within a single item), originality (quality of ideas, that can be reflected in uncommonness, cleverness, and remoteness of ideas), and elaboration (number of details provided along with the basic appropriate ideas; Giancola et al., 2023b; Guilford, 1967; Hocevar, 1979; Nijstad et al., 2010; Silvia et al., 2008). Under malevolent intentions, common scores for DT processes, such as originality and fluency, may not overlap with those observed in more traditional DT tasks. For instance, the potential to think divergently may prompt individuals to fluently generate malevolent ideas according to the degree to which individuals tend to inhibit their explicit report. Research showed that originality but not fluency as measured by the Alternative Uses Task (AUT)—a traditional paradigm in which individuals are asked to generate as many unconventional uses of everyday objects (e.g., a brick) as possible (Guilford, 1967)—predicted the generation of unsolicited malicious ideas in the same task (Dumas and Strickland, 2018). Perchtold-Stefan et al. (2021) explicitly instructed participants to generate malevolent ideas, and found both fluency and originality in a malevolent creativity task to be positively correlated; however, the authors (Perchtold-Stefan et al., 2021) found fluency but not originality in a separate, traditional DT task to be correlated with scores at the malevolent task. Thus, the directionality of the relationship between malevolence and DT processes remains unclear.

Despite the characteristics of the paradigms used may influence the way participants use their creative potential to prefer fluent, original, or malevolent ideation, recent neuroscientific findings revealed that similar topographical and temporal patterns of EEG alpha activations to those observed during general creative ideation support the creative generation of malevolent ideas (Perchtold-Stefan et al., 2023). Additionally, other studies showed that several brain areas are activated during both the generation of malevolent ideas and general creative thinking (Gao et al., 2022a). To further understand their commonalities, Gao et al. (2025) have analyzed brain activation and connectivity during the generation of creative ideas under malevolent and benevolent intentions. Their results show shared activation patterns during ideation, prominently in the middle and frontal gyri, whose activity is typically associated with high-order cognitive processing. Concurrently, the authors found distinct brain activity patterns, with greater activations and reduced connectivity in several brain areas related to, among others, attentional control (Gao et al., 2025). This evidence further suggests that common creative processes are deployed under malevolent or benevolent intentions, but also that distinct mechanisms may be involved. Therefore, exploring creativity from perspectives other than the underlying processes can be a fruitful approach to settle such differences and commonalities. Indeed, research has investigated creativity also concerning its products (e.g., artistic products) and through the assessment of individuals' acts during everyday life.

Research exploring creativity in terms of its products typically evaluates them according to their originality and appropriateness. Originality refers to creative products' novelty, uniqueness, and unconventionality, while appropriateness entails their effectiveness, utility, and context-dependent relevance (Runco and Jaeger, 2012; Acar et al., 2017). These two dimensions reflect the standard definition of creativity (Runco and Jaeger, 2012), although other models include additional attributes, such as aesthetics and elegance (Besemer and O'Quin, 1986). Creative products (e.g., a patent, an artwork, a scientific theory, an object) are evaluated by a panel of judges according to these attributes, typically using methodologies such as the consensual assessment technique (Amabile, 1982; Acar et al., 2017). As well as a process, assessing creativity as a product provides an important perspective for examining the malevolent use of creativity, for instance, in studying the contextual factors influencing malevolent innovation in terrorist attacks (Logan et al., 2023). Instead, as Runco (2010) proposed, the benevolent or malevolent nature of creativity is not determined by the potential to generate creative ideas or solutions but by their use and resulting products. Adopting a case-study methodology, Kapoor et al. (2016) highlighted the role of individual traits, attitudes, and environmental and contextual factors in defining originality and malevolence in products (Kapoor et al., 2016). Accordingly, one study (Harris and Reiter-Palmon, 2015) suggested that individual traits (implicit aggression and premeditation) and situational cues (benevolent or malevolent) interact in the emergence of original and malevolent solutions in a problem-solving task. While malevolent creativity in this area is less investigated, its contribution to the overarching understanding of the phenomenon would be high since it allows for verifying whether individual or contextual factors influencing the creative potential in terms of processes are also reflected in their tangible outcomes. In this regard, understanding which individual traits are related to the creative potential and also to its concrete transformation appears meaningful, especially when the product of such processes has malevolent intentions.

Concerning malevolence as reflected in creative behaviors during real-life acts (such as criminal or delinquent ones), it has been typically investigated through self-reported measures. Several measures of self-report creativity exist, focusing on exceptional or everyday creativity, assessing general or specific (e.g., arts or science) dimensions, and addressing public manifestations of creativity or self-beliefs (see Silvia et al., 2012). Malevolent behavior can be evaluated by self-reported questionnaires, such as the Malevolent Creativity Behavior Scale (MCBS, Hao et al., 2016), which is conceived as a measure of the tendency of individuals to generate malevolent ideas (e.g., deliberately hurting people, lying, playing tricks). This scale was found to predict the number of malevolent ideas generated in problem-solving tasks and to correlate with everyday general creative ideation scales, as assessed by the Runco Ideational Behavior Scale (Hao et al., 2016). Kapoor and Kaufman (2022b) further explored the link among self-reported creativity domains, malevolent creativity, and moral foundations. Their results pointed out that levels of binding moral foundations (purity, loyalty, and authority) were negatively associated with creativity (at least in the scholarly and art domains) and malevolent creativity, even though a mediation effect of dark personality traits was observed for the latter. In addition, the authors showed that individualizing moral foundations, i.e., care and fairness, positively predicted self-reported creativity (in the everyday, scholarly, and artistic domains) but was negatively associated with malevolent creativity. Overall, the evidence showed that there are many commonalities between general and malevolent creativity, but also that individual factors relating to personality and ethics can specifically promote harmful uses.

Research is fruitfully looking at cognitive styles to study the individual factors involved in creativity. In general, cognitive styles reflect stable and ubiquitous individual strategies of information acquisition and processing (Kozhevnikov, 2007). Among these, Field Dependence-Independence (FDI) has a long history of scientific speculation (Witkin, 1950) and still represents one of the most investigated cognitive styles (Mefoh et al., 2017). The construct of FDI refers to the disposition of individuals to be influenced by the surrounding context. According to Witkin et al. (1977), individuals lie on a continuum between field-dependence and field-independence: the latter reflects greater capability of inhibiting distracting information and focusing on processing target stimuli than dependent ones. For instance, several studies showed that field independence is positively associated with different facets of creativity, including DT (Lei et al., 2021; Li et al., 2020; Spotts and Mackler, 1967), production (Miller, 2007), self-report behavior (Fergusson, 1992, 1993), even though some studies presented mixed results (for a review based on creative process and product see Giancola et al., 2022a). Based on this three-fold perspective of creativity, the present study focused on the key role of FDI as the most potentially relevant cognitive style involved in creativity based on the current state of knowledge. Thus, the strength and originality of the present study is in assessing MC using a multifaceted approach (including process, product, and behavior). The concomitant examination of different components of MC associated with the aim of assessing the effects of field independence on creativity, after controlling for ethics, may provide a broad view of potentially relevant individual antecedents of MC.

Concerning DT, different studies have highlighted that field independents (FI) score higher than field dependents (FD) in multiple dimensions. For instance, using a brainstorming task, Li et al. (2020) have found FIs to show more fluency, novelty, and flexibility than FDs. Similarly, FI adolescents have been found to be more fluent and original (but not flexible) than their FD counterparts (Lei et al., 2021). In addition, using the AUT, FDI was found to moderate the effect of working memory capacity on DT in adolescents (Giancola et al., 2023a) and mediate the effect of fluid intelligence on DT in children (Giancola et al., 2024), adolescents (Giancola et al., 2023c), and young adults (Giancola et al., 2022c). Overall, these results highlight the involvement of cognitive styles in process-based creativity. FI individuals may fluently and flexibly generate more novel ideas in a short time than FDs, given their higher predisposition to analytical problem-solving and knowledge use.

Regarding creative production, FIs were found to outperform FDs (e.g., Miller, 2007). In a recent study by Giancola et al. (2022b), participants completed the Embedded Figure Test (EFT; Witkin et al., 1971) and performed a real-world creativity task, the Visual Creative Synthesis Task (Finke et al., 1992; Palmiero et al., 2015). Participants were first required to combine triads of visual components (pre-inventive phase) and then describe the functioning of their invention (inventive phase) according to a pre-defined category. The authors found that FIs ranked higher than FDs in all the pre-inventive (originality and synthesis) and inventive (originality and appropriateness) indicators. Within the Geneplore model framework (Finke et al., 1992), which explains creative production as occurring cyclically within a pre-inventive generative and inventive exploratory phase, the authors argued that FIs outperformed FDs in both phases, given their ability to shift between divergent and convergent styles of thinking (Giancola et al., 2022a).

The relationship between self-report behavior creativity and FDI has been scarcely investigated. Evidence of the relationship has been observed in two studies by Fergusson (1992, 1993), in which art students self-reported artistic ability positively but weakly correlated with field independence as measured by the EFT. However, given the highly specific creative field of these studies, generalization of the results is not possible, and further research employing standardized creativity measures of self-reported behavior is needed.

Although these findings suggest a consistent relationship between FDI and general creativity, no studies have examined the same relationship when considering the dark side of creativity. However, given the discussed commonalities in the dimensions of creativity, also creativity under malevolent intentions should be affected by FDI. As evidence reinforcing this link, previous research reported that FIs tend to exhibit a higher disposition toward hostile behaviors, manipulation, and opportunism than FDs (Witkin and Goodenough, 1977). In contrast, FDs tend to have a more positive outlook toward others. They are less likely to think or act in a malevolent way. They are described as friendly and polite, and they prefer to affiliate with others. They demonstrate a preference for interpersonal interactions and humanitarian interests (Witkin and Goodenough, 1977). Thus, FIs appear to be more self-focused than FDs. For instance, Kühnen et al. (2001) found that priming participants with independent self-knowledge induced more field-independence on the EFT than priming them with interdependent self-knowledge. Relatedly, field independence on the EFT was found to be correlated with the narcissistic personality trait (Konrath et al., 2009), which is also one of the major predictors of malevolent creativity (Dow, 2023; Zhou et al., 2024). Taken together, this evidence supports the hypothesis that FIs are more prone to exhibit high malevolent creativity.

Notably, as the impact of FDI on malevolent DT, production, and behavior can be affected by several individual factors, in the present study, socio-demographics (e.g., Dumas and Strickland, 2018; Harris and Reiter-Palmon, 2015) and social desirability (as discussed in several MC studies, e.g., Hao et al., 2016; Szabó et al., 2022) were included as controlling variables. As for sociodemographic factors, some studies found that gender plays a role in malevolent creativity. Indeed, some studies suggested that men exhibited higher malevolent creativity than women in several studies (Dumas and Strickland, 2018; Harris and Reiter-Palmon, 2015; Perchtold-Stefan et al., 2021). Traditionally, such a result has been explained according to the evidence that men manifest more aggressive tendencies than women (Lee and Dow, 2011), even though other results suggest opposite views. For instance, Kapoor (2019) found no differences in mean negative creativity between men and women across four experiments and argued for a gender similarity hypothesis, stating that gender differences may rely upon cognitive styles used when solving creative tasks and that the task content is crucial (e.g., socially oriented) for the differences to emerge. For these reasons, given the objectives of the present study, controlling for gender appears straightforward. Further evidence in general creativity found a role of age (e.g., Palmiero, 2015; Fusi et al., 2021) and education level (e.g., Palmiero, 2015; Palmiero et al., 2017). Concerning age, a cross-sectional study by Palmiero (2015) has shown that divergent thinking and creative production abilities peak before the age of 40 years before undergoing domain-specific stabilization (see Fusi et al., 2021 for a review). Interestingly, in the same study (Palmiero, 2015) and another study (Palmiero et al., 2017), a significant effect of the education covariate was found, even though a cohort effect might have generated it. Therefore, although no specific studies are focusing on the relationship between these variables and MC, we chose to control for them in the present work. We also included a measure of the social desirability bias since, as is already pointed out (e.g., Hao et al., 2016), it may discourage individuals from explicitly expressing malevolent creative ideas due to self-deception and impression management (Paulhus and Reid, 1991).

Additionally, it has been shown that mood and emotional states on the one hand (Baas et al., 2019; Perchtold-Stefan et al., 2021) and ethical tendencies on the other (e.g., Kapoor and Kaufman, 2022a,b; Storme et al., 2021) may strongly influence malevolent creativity. For instance, a recent study showed that anger mediates the relationship between sensitivity to injustice and malevolent creative behavior, as individuals more prone to perceive injustice tend to report a higher level of anger and, in turn, more malevolent creative behavior (Wang et al., 2024). Given the role of mood and ethicality in both general and malevolent creativity, we treated them as additional potential confounding variables.

As for the association between mood, emotional state, and creativity, different studies showed inconsistent results. Evidence has shown that positive and negative moods may influence creative ideation, even though their effects are shown to be context-dependent (Palmiero et al., 2023; Davis, 2009 for a meta-analysis). In addition, research pointed out the key role of negative emotions in malevolent creativity, even though few studies are available. Perchtold-Stefan et al. (2021) administered participants with an MC task, in which they were asked to write original ideas about how to deal with a negative social situation in a malevolent way (e.g., taking revenge) and rated their current angry mood at the beginning of the experiment. They found that state anger levels were positively correlated with performance in the MC task. Previously, Baas et al. (2019) investigated the effects of social threat on MC, comparing a high- vs. a low-socially threatening prisoner's dilemma task. They administered an AUT and found that the number of neutral original ideas did not differ between social threat conditions. Interestingly, the number of malevolent original ideas was higher in participants experiencing the social threat condition. Both results have been interpreted in terms of valence congruency. In one case, the congruency is between the harmful materials presented within the task to perform and the current mood (Perchtold-Stefan et al., 2021). In the other study, it is conceived as an explaining mechanism of heightened attentional processing toward emotionally congruent cues driven by the experience of social threat, resulting in the increased creative generation of intentionally harmful ideas (Baas et al., 2019). Thus, state emotions seem to direct cognitive processes toward the creative generation of compatible outcomes.

Concerning the link between ethicality and creativity, Storme et al. (2021) found evidence that creative individuals generally tend to engage in unethical behavior more than non-creative individuals. The commonalities between creativity and unethicality have been proposed to be a strong sense of entitlement, a more remarkable ability to justify unethical behavior, and a tendency toward non-conformity (Storme et al., 2021). However, such a link is not straightforward. For instance, others have proposed that individuals use compensatory strategies to reduce the ethical dissonance arising from immoral actions (Barkan et al., 2012; Jordan et al., 2011). In this sense, ethical tendencies, such as prosocial behavior, can also be the byproduct of creativity through the moral regulation of ethical dissonance. Relatedly, studies have found that engaging in moral reasoning suppresses creative malevolent behavior and ideation (Fu and Zhang, 2022; Zhao et al., 2022).

Furthermore, other studies suggested the involvement of dark personality traits, characterized by low ethicality, in malevolent creativity (Gao et al., 2022b; Kapoor and Kaufman, 2022b), even though the results are contrasting when task-based measures of malevolent creativity are employed (Kapoor et al., 2024; Kapoor and Khan, 2016). Notably, as Forsyth (1992) and Forsyth et al. (2008) suggested, ethical positions can be described according to two orthogonal dimensions: idealism—i.e., the degree of concern of benevolent outcomes of one's action—and relativism—i.e., the degree of acceptance of universal moral principles. Interestingly, one study found that self-reported general creativity positively predicted both (Bierly et al., 2009), suggesting that creative individuals are characterized by skepticism about universal moral rules (high relativism) and social sensitivity and ethics of caring (high idealism), making them *situationists* using Forsyth et al.'s (2008) dimensions. Concerning the malevolent use of creativity, it is reasonable to think that individuals with higher malevolent creativity behaviors exhibit low idealism since they are supposed to be less sensitive to caring for others and having positive outcomes. Thus, following Forsyth et al. (2008), malevolent creativity may be associated with an egoistic subjectivist moral viewpoint.

## 2 The present study

The main objective of the present study was to investigate whether FDI as an individual cognitive style can predict malevolent creativity as measured in terms of DT, product, and self-reported behaviorafter controlling for demographics (age, gender, and education), social desirability, state mood and ethical positions (idealism and relativism). In line with results on general creativity, field-independent individuals were expected to get higher scores on all three malevolent measures than field-dependent ones.

The hypotheses were formulated as follows:

– (H1—process hypothesis) FI individuals generate more malevolent ideas than FDs when engaged in DT.
– (H2—product hypothesis) FIs produce more malevolent creative products than FDs.
– (H3—self-reported behavior hypothesis) FIs report higher malevolent self-report creative behavior than FDs.

## 3 Methods

### 3.1 Participants and procedure

A total of 201 individuals participated in the study. Participants reporting a history of brain lesions, psychiatric disorders, current use of psychopharmacological medications were excluded from the analysis (*N* = 7). In addition, three participants were excluded because they did not fill out at least one measure or did not provide relevant sociodemographic information. The final sample consisted of 191 participants (*M* age = 22.8, *SD* age = 2.3; 65 men and 126 women). An a priori Power Analysis conducted in G^*^Power 3.1.9.7 (Faul et al., 2009) showed that at least 160 participants were sufficient to detect a medium effect size of *f*^2^= 0.15 with a power of 0.95 and an α of 0.05 in a linear multiple regression model with eight predictors. Participants were tested individually. First, they were informed about the experiment and invited to sign the informed consent form. Afterward, they were administered all the measures in randomized order. Hereafter, a description of each measure is provided. The study was approved by the Internal Review Board of the University of L'Aquila (Prot n. 39870 of 30/04/2020), and all participants provided written informed consent for participation. The study was conducted in accordance with the principles of the Declaration of Helsinki.

### 3.2 Materials

#### 3.2.1 Independent variable

Field Dependence-Independence was assessed through the *Embedded Figure Test* (EFT; Witkin et al., 1971; Italian version, Series A, Fogliani et al., 1984), in which participants were required to detect a simple black and white shape embedded within a more complex colored figure. The test is made up of 12 trials. The complex figure was presented in each trial for 15 s, and the participants had to describe it. Then, the figure disappears and is replaced by the simple one. After 10 s, the complex figure was present again, and participants had to localize it and draw the outline of the simple figure with a pencil. The response time was recorded until participants provided the correct response or 180 s elapsed. The average response time was taken as a measure of field dependency, where the shorter the time, the higher the predisposition toward field independence. The test had excellent reliability (Cronbach's alpha = 0.91).

#### 3.2.2 Dependent variables

The process-based malevolent creativity was assessed using a modified version of the AUT (Guilford, 1967). In this adapted version, participants were instructed to generate original and intentionally harmful (i.e., malevolent) uses for three everyday objects: a brick, a fork, and a shovel. The instructions explicitly encouraged the production of ideas aimed at causing harm or damage. Each object was presented separately, and participants were given 2 min per object to generate as many different malevolent uses as possible. This version of the AUT served as a process-based measure of malevolent creativity. Two trained independent judges (2 females) were asked to score the individual protocols in terms of creative malevolence. Specifically, an adaptation of the snapshot method was used (see Silvia et al., 2008): for each subject, judges were instructed to give independently a single holistic rating to all alternative uses provided, weighing the attributes of uncommonness, remoteness, and cleverness, from 1 (not at all creative) to 5 (highly creative). Then, the final score was the average of the scores provided by the two judges. The intraclass correlation coefficient (ICC) described an excellent inter-rater agreement (*ICC* = 0.96, *p* < 0.001).

The product-based malevolent creativity was evaluated using the *Cartoon Caption Task* (CCT; Nusbaum et al., 2017). This task required to write a caption for five The New Yorker cartoons, such as (1) an astronaut on the moon talking on a cell phone, (2) Batman and Superman sitting on a sofa talking to a psychotherapist, (3) a pirate talking to the crew and showing three boxes of rockets on the boat; (4) a woman detective and a policeman looking at a human shape depicted on the floor; and (5) a crazy wife and an amazed husband having breakfast. Participants were requested to provide an offensive caption for each cartoon in 2 min. For each caption, two independent judges (2 females) were asked to evaluate the degree of creative malevolence from 1 = not at all to 5 = at all. The *ICC* varied between 0.796 (95% *CI* 0.737–0.842) and 0.894 (95% *CI* 0.862–0.920), indicating high agreement between the two raters (*p* < 0.001). The final score was averaged between the two raters across the five captions.

Self-reported creativity was assessed through the Malevolent *Creativity Behavior Scale* (MCBS; Hao et al., 2016). The questionnaire consists of 13 items describing malicious behaviors occurring in everyday life, such as hurting people, lying, and playing tricks. Responses were scored on a 5-point Likert scale ranging from never (0) to usually (4). The sum of the ratings was taken as the MCBS score, with higher scores indicating a higher tendency to malevolent creativity behaviors. In the present study, the internal consistency reliability was high with Cronbach's alpha = 0.81).

#### 3.2.3 Controlling variables

Participants were requested to fill in a brief sociodemographic questionnaire on age, gender (coded as male = 1, female = 0), educational level (in years), history of brain lesions, diagnosis of psychiatric disorders, and drugs/alcohol and medication use.

The *Social Desirability Scale* (Manganelli Rattazzi et al., 2000) was used to measure the participants' tendency to present themselves in a positive light. The questionnaire consists of nine items on a 9-point Likert scale. Item scores were summed up to measure the extent to which participants tended to present themselves favorably. The scale's reliability in the present sample was good (Cronbach's alpha = 0.71).

Through the *Profile of Mood States* (Farnè et al., 1991), participants rated mood descriptions presented in each of the 65 items on a 5-point Likert scale ranging from not at all to extremely (0–4). The total mood disturbance score was calculated as the sum of the five negative subscales (Tension, Depression, Anger, Fatigue, Confusion) minus the positive subscale (Vigor). The internal consistency reliability was excellent, with Cronbach's alpha = 0.96.

The *Ethics Position Questionnaire* (O'Boyle and Forsyth, 2021) was administered to evaluate the two ethical positions of Idealism and Relativism. Participants reported their agreement with ethical sentences through a 5-point Likert scale ranging from strongly disagree (1) to strongly agree (5) in each of the five items composing the two subscales (Idealism and Relativism). Item scores were summed separately for the two subscales. In the present study, both scales had high internal consistency reliability with Cronbach's alpha = 0.90 and 0.80 for the Idealism and Relativism subscales, respectively.

### 3.3 Statistical analyses

Separate hierarchical linear regression analyses with five blocks were conducted to analyze the relative contribution of the factors predicting each of the three malevolent creativity measures obtained in the present study (AUT, CCT, MCBS). We were indeed interested in assessing the proportion of unique variance explained by the predictors in malevolent creativity, especially FDI, controlling for all the other factors. In the first block, sociodemographic variables (age, gender, and years of education) were included. According to the literature, as the explicit expression of MC may be driven by the participants' concerns about social desirability, their current state mood, and their ethical positions, these variables were included sequentially to verify the overall contribution of each new block of variables. Thus, the second block included social desirability in the model. Then, mood disturbance was inserted in the third block to evaluate the impact of state emotional negativity over MC, and was followed by the two ethical subscales (Idealism and Relativism) to examine their role above potential situational differences in state mood in the fourth step. Finally, the EFT score, as our variable of interest, was entered in the last block to assess the unique contribution of this and the other variables after controlling for the effect of all the others. Descriptive statistics are reported in Table 1. Age, education years, and all the scores were converted to *z*-scores before being analyzed. Then, the normality of the distribution of the scores of three dependent variables was tested by applying *z*-tests using skewness (0.69, 0.50, and −0.19 for MCBS, CCT, and DT, respectively; *SE* = 0.18) and kurtosis (0.31, 0.87, and −0.65 for MCBS, CCT, and DT, respectively; *SE* = 0.35). Since the MCBS score turned out to violate normality (*Z*_skewness_ > 3.29; Kim, 2013), it was log-transformed (1 was added to the raw scores) before being *z*-transformed. Statistical significance was set at *p* < 0.05.

**Table 1:** Descriptive statistics.

**Variable**	**Mean**	**SE**	**Min**	**Max**
Age	22.79	0.16	18	31
Education years	15.1	0.18	5	20
Social desirability	34.11	0.49	19	54
Mood disturbance	37.3	2.5	−16	144
Idealism	21.28	0.31	5	25
Relativism	15.99	0.34	5	25
EFT	47.87	3.14	5	180
MC—Process (DT)	3.12	0.09	0	5
MC—Product (CCT)	1.54	0.04	0	3.4
MC—Behavior (MCBS)	14.64	0.51	0	38

N = 191.

EFT, Embedded Figure Test (in seconds); DT, Divergent thinking; CCT, Cartoon Caption Task; MCBS, Malevolent Creativity Behavior Scale.

## 4 Results

### 4.1 Malevolent creativity—Process

Age, gender, and years of education together explain 10.3% of the variance in DT, *adj*.*R*^2^ = 0.088, *F*_(3, 187)_ = 7.14, *p* < 0.001. The inclusion of social desirability in block 2 contributed to a significant increment of 2.6% of variance explained, *Fchange*_(1, 186)_ = 5.61, *p* = 0.019. The predictors explain 12.9% of the variance in DT, *adj*.*R*^2^ = 0.11, *F*_(4, 186)_ = 6.89, *p* < 0.001. In Block 3, the inclusion of mood disturbance provided a significant increment of 2% of explained variance, *Fchange*_(1, 185)_ = 4.34, *p* = 0.039, yielding a total explained variance of 14.9%, *adj*.*R*^2^ = 0.13, *F*_(5, 185)_ = 6.482, *p* < 0.001. In Block 4, Idealism and Relativism added no statistically significant contribution to the explained variance, Δ*R*^2^ = 0.002, *Fchange*_(2, 183)_ < 1, with a total explained variance of 15.1%, *adj*.*R*^2^ = 0.119, *F*_(7, 183)_ = 4.66, *p* < 0.001. Finally, the EFT added a significant contribution of 6.5% explained variance, *Fchange*_(1, 182)_ = 15.027, *p* < 0.001, with all the predictors accounting for the 21.6% of explained variance, *adj*.*R*^2^ = 0.182, *F*_(8, 182)_ = 6.27, *p* < 0.001. Collinearity (VIF) was within acceptable levels (Table 2), and the Shapiro-Wilk test did not reveal a violation of the normality assumption on the residuals (*W* = 0.99, *p* = 0.091).

**Table 2:** Regression coefficients for each step-5 model.

**Predictor**	***B***	**95% CI B**		***p***	**VIF**
		**Lower**	**Upper**		
**Process—Malevolent DT (*****N*** = **191)**					
Intercept	−0.173	−0.336	−0.009	0.038^*^	
Gender (*m* = 1)	0.507	0.214	0.800	< 0.001^***^	1.155
Age	0.084	−0.065	0.233	0.269	1.329
Education years	0.081	−0.066	0.227	0.277	1.279
Social desirability	−0.08	−0.223	0.063	0.273	1.223
Mood disturbance	0.168	0.036	0.300	0.013^*^	1.036
Idealism	−0.009	−0.152	0.135	0.907	1.234
Relativism	0.04	−0.094	0.174	0.556	1.064
EFT	−0.27	−0.407	−0.132	< 0.001^***^	1.124
**Product—Malevolent CCT (*****N*** = **191)**					
Intercept	−0.163	−0.324	−0.002	0.047^*^	
Gender (*m* = 1)	0.479	0.191	0.768	0.001^**^	1.155
Age	0.097	−0.05	0.244	0.194	1.329
Education years	0.134	−0.01	0.278	0.068	1.279
Social desirability	−0.187	−0.328	−0.046	0.01^*^	1.223
Mood disturbance	−0.012	−0.141	−0.118	0.859	1.036
Idealism	−0.113	−0.255	0.029	0.117	1.234
Relativism	0.101	−0.031	0.232	0.133	1.064
EFT	−0.178	−0.313	−0.043	0.01^*^	1.124
**Self-reported behavior—MCBS (*****N*** = **188)**					
Intercept	−0.045	−0.174	0.084	0.494	
Gender (*m* = 1)	0.317	0.087	0.548	0.007^**^	1.154
Age	−0.08	−0.198	0.037	0.18	1.323
Education years	0.03	−0.085	0.145	0.608	1.278
Social desirability	−0.205	−0.319	−0.090	< 0.001^***^	1.205
Mood disturbance	0.179	0.075	0.283	< 0.001^***^	1.039
Idealism	−0.087	−0.203	0.030	0.144	1.237
Relativism	0.133	0.028	0.239	0.013^*^	1.052
EFT	−0.066	−0.179	0.047	0.253	1.122

^*^p < 0.05.

^**^p < 0.01.

^***^p < 0.001.

EFT, Embedded Figure Test; DT, Divergent thinking; CCT, Cartoon Caption Task; MCBS, Malevolent Creativity Behavior Scale; VIF, Variance Inflation Factor.

**Figure 1:**
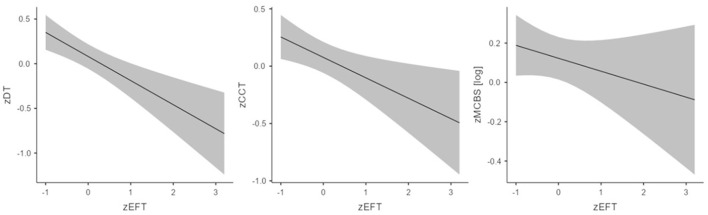
The effect of FDI on each of the three MC variables. EFT, Embedded Figure Test; DT, Divergent Thinking **(left)**; CCT, Cartoon Caption Task **(middle)**; MCBS, Malevolent Creativity Behavior Scale (log-transformed) **(right)**. All variables are standardized (z-scores). Shaded areas denote 95% Confidence Intervals.

The coefficients for each predictor within the final block of the regression analysis are reported in Table 2. The EFT mean response time significantly and negatively predicted performance in the DT task, meaning that the more FI individuals were (as indicated by lower EFT mean response time), the higher the malevolent DT score (Figure 1). Furthermore, mood disturbance was significantly and positively associated with performance in the DT task, linking higher scores with a higher degree of mood disturbance. Finally, we found a significant effect of gender, given that men obtained higher DT scores than women.

### 4.2 Malevolent creativity—Product

The sociodemographic variables included in block 1 jointly explained 13.1% of the variance in the CCT performance, *adj*.*R*^2^ = 0.12, *F*_(3, 187)_ = 9.37, *p* < 0.001. Adding the social desirability score in block 2 increased significantly explained variance by 6.4%, *Fchange*_(1, 186)_ = 14.84, *p* < 0.001. Together, the predictors in block 2 explained 19.5% of the variance, *adj*.*R*^2^ = 0.18, *F*_(4, 186)_ = 11.261, *p* < 0.001. The inclusion of mood disturbance and the ethical variables did not significantly increase explained variance, Δ*R*^2^ = 0.001, *Fchange*_(1, 185)_ < 1 and Δ*R*^2^ = 0.016, *Fchange*_(2, 183)_ = 1.89, *p* = 0.154 for the comparison between model 3 and model 2, and between model 4 and model 3, respectively. The proportion of explained variance was 19.6%, *adj*.*R*^2^ = 0.174, *F*_(5, 185)_ = 8.99, *p* < 0.001 at step 3 and 21.2%, *adj*.*R*^2^ = 0.182, *F*_(7, 183)_ = 7.030, *p* < 0.001 at step 4. The inclusion of the EFT score in the last step increased significantly the explained variance of 2.8%, *Fchange*_(1, 182)_ = 6.77, *p* = 0.01. Total variance explained in step 5 by all the predictors was 24%, *adj*.*R*^2^ = 0.21, *F*_(8, 182)_ = 7.19, *p* < 0.001. Collinearity (VIF) was within acceptable levels (Table 2), and the Shapiro-Wilk test did not reveal a violation of the normality assumption on the residuals (*W* = 0.99, *p* = 0.501).

The coefficients for the predictors included in the final model are reported in Table 2. The EFT mean response time significantly and negatively predicted malevolent creative production, i.e., the more FI individuals were (lower EFT mean response time), the higher the CCT score (Figure 1). In addition, social desirability was significantly and negatively related to product malevolent creativity, linking lower CCT scores with a higher tendency to present themselves in a favorable light. We also find a significant effect of gender, given that men obtained higher CCT scores than women.

### 4.3 Malevolent creativity—Self-reported behavior

Concerning the MCBS score, we excluded observations (*N* = 3) with values exceeding four *z*-scores from the regression analysis. The first model, which included age, gender, and years of education explained 7.7% of the variance, *adj*.*R*^2^ = 0.06, *F*_(3, 184)_ = 5.08, *p* = 0.002. The inclusion of social desirability sharply and significantly increased explained variance of 9.6%, *Fchange*_(1, 183)_ = 21.19, *p* < 0.001, with all four predictors together explaining 17.2% of the variance in MCBS, *adj*.*R*^2^ = 0.15, *F*_(4, 183)_ = 9.53, *p* < 0.001. The inclusion of mood disturbance also significantly increased the variance explained, but to a lesser extent, Δ*R*^2^ = 4.4%, *Fchange*_(1, 182)_ = 10.29, *p* = 0.002. The proportion of explained variance in step 3 was 21.7%, *adj*.*R*^2^ = 0.195, *F*_(5, 182)_ = 10.07, *p* < 0.001. In step 4, the inclusion of Idealism and Relativism significantly added 3.1% of explained variance, *Fchange*_(2, 180)_ = 3.76, *p* = 0.025, with a total variance explained of 24.8%, *adj*.*R*^2^ = 0.22, *F*_(7, 180)_ = 8.48, *p* < 0.001. Finally, in the last step, including the EFT produced a non-significant change in the explained variance, Δ*R*^2^ = 0.005, *Fchange*_(1, 179)_ = 1.32, *p* = 0.253. The total variance explained was 25.4%, *adj*.*R*^2^ = 0.22, *F*_(8, 179)_ = 7.60, *p* < 0.001. Collinearity (VIF) was within acceptable levels (Table 2), and the Shapiro-Wilk test did not reveal a violation of the normality assumption on the residuals (*W* = 0.997, *p* = 0.956).

The predictors' estimates within model 5 are reported in Table 2. The MCBS score was not significantly predicted by the EFT (Figure 1), but it was significantly and positively predicted by Relativism and mood disturbance: higher self-reported MC behavioral tendencies in everyday situations were associated with both higher scores in relativism and higher mood disturbance levels. In addition, social desirability was significantly and negatively related to MCBS, linking lower MCBS scores with a higher tendency to present themselves in a favorable light. Even in this case, there was a significant effect of gender, given that men obtained higher MCBS scores than women.

## 5 Discussion

The present study aimed to investigate the influence of FDI on the disposition toward malevolence in DT, creative production, and self-reported creativity after controlling for demographics, social desirability, state mood, and ethical positions. Although research into the field of creativity showed that FDI plays a role in different creativity dimensions (Cropley, 1997; Li et al., 2020), no studies thus far explored the same relationship with regard to the dark side of creativity.

Concerning process creativity, we observed that the primary variable of interest, FDI, significantly affected malevolent DT. Specifically, we found that the more independent individuals were, the more likely they were to generate malevolent original ideas in the AUT. Similarly, we found that product-creativity was also significantly predicted by FDI. Indeed, we found that the field-independent individuals were more prone to produce original but malevolent cartoon captions. Therefore, the hypotheses H1 and H2 are supported by our results. These results also support previous research on the link between field independence and creativity, further suggesting the commonalities between the mechanisms underlying general and malevolent creativity.

As in the literature on general creativity, the influence of FDI on creative tasks can be traced back to the evidence linking field-independent individuals with greater flexibility and a more remarkable ability to overcome habitual and routine behaviors and thoughts (Giancola et al., 2022b, 2023a). For instance, field-independent individuals are generally better than field-dependent ones in analytic thinking and problem-solving tasks (Mefoh et al., 2017). Field-independent individuals also generally outperform field-dependent individuals in both visual and verbal creativity tasks (Giancola et al., 2022b; Lei et al., 2021), possibly given their ability to minimize the impact of irrelevant external information (Yao and Diao, 2024) and to shift between divergent and convergent thinking styles (Giancola et al., 2022a,b). These abilities are particularly evident when considering the process- and product-based dimensions of creativity. In the alternate uses task (AUT), individuals are asked to retrieve their knowledge concerning the use of an object and update it to favor the generation of novel and appropriate uses of the same objects in a continuous process of cognitive reorganization. In this sense, the increased capacity to process relevant information and inhibit irrelevant information of field-independent individuals puts them in an advantageous position over field-dependent ones in divergent thinking tasks. Thus, it is plausible that the ability of field-independent individuals also extends to malevolent creativity, which is defined in terms of both process and product. This would support the hypothesis that the role of field independence in creativity can be comprehensive and not tied to specific aspects. FIs are likely more task-oriented than FDs, allowing them to prioritize their knowledge over external influences, which could support their creative processes and inventiveness, even for malevolent outputs.

Besides, field independence might be tied to malevolent creativity, considering the social dimensions embedded in creative tasks. Li et al. (2020) showed that even though FIs performed better than FDs in DT during a brainstorming task, no difference was found in terms of novelty when participants had the opportunity to collaborate. This means that when information is shared, FDs may exhibit creative ideation, given their tendency to rely heavily on environmental and social cues to process relevant evidence. In this vein, while FIs are generally more autonomous and less susceptible to others' opinions, they also exhibit more emotional and physical distance from others than FDs (Kline et al., 1984; Witkin and Goodenough, 1977). Such a reduced interest in others might increase the tendency toward the malicious expression of creativity. Contrary to this, FDs tend to conform to social norms and group expectations, resulting in reduced negative creativity. In this regard, considering social desirability, we found that while it added a significant contribution over sociodemographic variables in both process- and product-creativity models, it only predicted malevolent creative production after controlling for all the other variables. In this case, it is reasonable to think that participants who are less concerned with social desirability produced more malevolent captions compared to those who tended to describe themselves in a positive light. In contrast, malevolent DT may be less sensitive to social desirability. On the contrary, we found that mood disturbance significantly influenced malevolent DT but not malevolent creative production. Coherently with previous literature (Baas et al., 2019; Perchtold-Stefan et al., 2021), the first result highlights that individuals with higher mood disturbance scores obtained higher scores in the malevolent AUT, confirming that negative emotions may heighten attentional sensitivity to harmful ideas. Instead, the association between malevolent creative production and mood might have been concealed by the humoristic component of the Cartoon Caption Task, hiding the effects of the emotional congruence between one's mood state and the malevolent task instructions.

We also found that FI does not predict self-reported malevolent creativity behavioral tendencies in everyday situations, as assessed by the MCBS. Thus, H3 was not supported. This may be partially explained in terms of the nature of the measures used to assess FDI and MC. Unlike the other two performance-based creative measures, the MCBS is self-reported, and it might not have been able to capture variations due to the cognitive style of interest. In this regard, social desirability and relativism predicted the MCBS score, suggesting that individuals with low concerns about social desirability and high relativism reported less malevolent creative behavioral tendencies. The role of these self-reported variables on the explicit reporting of malevolent creativity might have concealed any effect of FDI. However, we observed significant effects of mood on self-reported MC behaviors. The negative mood might have driven participants to perceive themselves as acting more maliciously in everyday situations. In addition, while ethical positions did not account for performance-based measures, we observed a significant effect on self-reported everyday MC. Indeed, it can be argued that the degree of malevolence associated with the former can be perceived by individuals as more situational and less concerning the stable description of their attitude or personality. Specifically, we observed that participants who were highly relativist were more prone to engaging in malevolent behaviors. This is in line with the view that creative individuals show high degrees of relativism (Bierly et al., 2009) and are unbound to universal moral foundations, rejecting conformity and not adhering to pre-determined general rules of behavior. Regarding idealism, no significant difference was found, suggesting that while high idealism is tied to general (benevolent) creativity (Bierly et al., 2009), such a link may vanish when considering its malicious dimensions.

Finally, concerning the sociodemographic variables, consistently with several other results an effect of gender was found (Dumas and Strickland, 2018; Harris and Reiter-Palmon, 2015; Perchtold-Stefan et al., 2021), with men obtaining higher scores than women in each of the three MC measures, supporting the traditional view that men are more prone to explicit aggressive ideas (Lee and Dow, 2011; but see Kapoor, 2019). Although the role of gender in MC needs to be further clarified, our results confirm the importance of controlling for this factor in studies investigating this construct. Differently, we did not find significant effects of age and education on any of the three dependent variables. While the literature has shown that these influence general creativity (e.g., Palmiero, 2015; Fusi et al., 2021), the lack of an effect in the present study might be interpreted in terms of the restricted age range of our sample. Therefore, cross-sectional studies are needed to investigate whether MC is influenced by these variables, such as general creativity.

### 5.1 Limitations

The present study shows several limitations that open up future research avenues. First, malevolent creative behaviors were evaluated using a self-report questionnaire (the MCBS), which does not fully reflect malevolent behavior in an ecological context. Future studies should employ more ecologically valid measures of malevolent creative behaviors, potentially integrating self-report with other-report measures. Second, only verbal measures of MC were included. Future studies should provide a more comprehensive evaluation of creativity and employ visual measures of malevolent creativity. Third, even though FDI represents a critical factor in explaining individual differences in creativity, a more granular evaluation of the role of cognitive styles in malevolent creativity should be envisaged, including a wide array of cognitive styles. Fourth, our sample was not equally distributed in terms of gender. Future research should confirm our results, considering a more balanced sample, taking into account that women generally display greater ethical inclinations and social desirability than men (Cropley et al., 2014), which may affect individual malevolent creative performance.

## 6 Conclusions

Taken together, the results of the present study showed that cognitive styles—field dependence/independence—play a role in the generation of malevolent creative ideas, both in process- and product-based measures of malevolent creative performance. Namely, field-independent individuals outperformed field-dependent individuals, possibly due to their greater flexibility and ability to process relevant information and lesser reliance on environmental and social cues. However, we found FDI not to be associated with self-reported MC behavioral tendencies in everyday life. The present results have been observed while controlling for known predictors of MC, like demographics, social desirability, state mood, and ethical positions. The findings corroborate those observed in the field of general creativity, proving that field independence has a comprehensive influence on creative processes, whatever the valence of their aims. Further research could investigate whether it is the processing of problem-related information or that of social cues that differentiates field independents and field dependents in general and malevolent creativity tasks, and the extent of such an influence in everyday situations. Finally, these results can be highly applicable in fields pertaining to the identification of malevolent creativity based on the characteristics of individuals. In addition, the results suggest potential applications in educational settings, providing valid foundations for the individuals' positive development. Cognitive styles can be used for the early detection of the malevolent use of creativity, becoming paramount factors in the definition of interventions capable of suppressing the generation or speeding up the decay of harmful creative ideas.

## Data Availability

The raw data supporting the conclusions of this article will be made available by the authors, without undue reservation.
